# A Novel Hyperactive Nud1 Mitotic Exit Network Scaffold Causes Spindle Position Checkpoint Bypass in Budding Yeast

**DOI:** 10.3390/cells11010046

**Published:** 2021-12-24

**Authors:** Michael Vannini, Victoria R. Mingione, Ashleigh Meyer, Courtney Sniffen, Jenna Whalen, Anupama Seshan

**Affiliations:** 1 Boston University School of Medicine, Boston, MA 02118, USA; vanninim@bu.edu; 2 Department of Pharmacological Sciences, Stony Brook University School of Medicine, Stony Brook, NY 11794, USA; victoria.mingione@stonybrook.edu; 3 Dana Farber Cancer Institute, Boston, MA 02215, USA; ashleigh_meyer@dfci.harvard.edu; 4 Renaissance School of Medicine, Stony Brook University Hospital, Stony Brook, NY 11794, USA; courtney.sniffen@stonybrookmedicine.edu; 5 Department of Molecular, Cell, and Cancer Biology, University of Massachusetts Medical School, Worcester, MA 01605, USA; jenna.whalen@umassmed.edu; 6 Department of Biology, Emmanuel College, Boston, MA 02115, USA

**Keywords:** Nud1, Cdc15, MEN, mitotic exit, Dbf2, Mob1, spindle position checkpoint

## Abstract

Mitotic exit is a critical cell cycle transition that requires the careful coordination of nuclear positioning and cyclin B destruction in budding yeast for the maintenance of genome integrity. The mitotic exit network (MEN) is a Ras-like signal transduction pathway that promotes this process during anaphase. A crucial step in MEN activation occurs when the Dbf2-Mob1 protein kinase complex associates with the Nud1 scaffold protein at the yeast spindle pole bodies (SPBs; centrosome equivalents) and thereby becomes activated. This requires prior priming phosphorylation of Nud1 by Cdc15 at SPBs. Cdc15 activation, in turn, requires both the Tem1 GTPase and the Polo kinase Cdc5, but how Cdc15 associates with SPBs is not well understood. We have identified a hyperactive allele of *NUD1*, *nud1-A308T*, that recruits Cdc15 to SPBs in all stages of the cell cycle in a *CDC5*-independent manner. This allele leads to early recruitment of Dbf2-Mob1 during metaphase and requires known Cdc15 phospho-sites on Nud1. The presence of *nud1-A308T* leads to loss of coupling between nuclear position and mitotic exit in cells with mispositioned spindles. Our findings highlight the importance of scaffold regulation in signaling pathways to prevent improper activation.

## 1. Introduction

Mitotic cyclins, such as cyclin A and cyclin B, complexed with cyclin-dependent kinases (CDKs), are necessary and sufficient for cells to enter the mitotic phase of the cell division cycle [1]. Therefore, it follows that exit from mitosis and preparation for the subsequent cell cycle requires the inactivation of mitotic cyclin–CDK complexes [2]. This is achieved in large part by the destruction of mitotic cyclins during anaphase through ubiquitin-mediated proteolysis initiated by the anaphase promoting complex or cyclosome (APC/C) ubiquitin ligase [3,4,5,6]. In budding yeast, the essential phosphatase Cdc14 triggers the ubiquitination of Clb2, the cyclin B homolog, by the APC/C and its late mitotic activator Cdh1. Cdc14 dephosphorylates Cdh1, which leads to Cdh1-APC/C activation. Cdc14 also promotes the accumulation of the mitotic CDK inhibitor Sic1 to trigger the exit from mitosis [7,8,9]. 

The regulated activation of Cdc14 is critical for both cell cycle progression and the maintenance of proper ploidy. In the absence of Cdc14 activation, cells remain arrested in anaphase with elongated spindles and high Clb2 levels [7,8,9]. On the other hand, in cells where Cdc14 is activated prematurely during the cell cycle by *GAL*-induced overexpression, exit from mitosis also occurs prematurely [8]. The timing of Cdc14 activation is particularly crucial for mitotic checkpoints, such as the spindle assembly checkpoint (SAC) and the spindle position checkpoint (SPoC), to function properly. The SAC is required to restrain cell cycle progression during the metaphase-to-anaphase transition until all chromosomes have successfully attached to mitotic spindle microtubules in a bipolar manner, which prevents errors in chromosome segregation and aneuploidy [10]. The SPoC functions in anaphase to ensure the coupling of nuclear position and mitotic exit. In cells with mispositioned spindles, where anaphase occurs entirely within the mother cell, the SPoC is essential to preserve ploidy in the daughter cells [11,12]. Cdc14 activation is controlled by a Ras-like signaling pathway known as the mitotic exit network (MEN). The core MEN components that function upstream of Cdc14 include the small GTPase Tem1, the Hippo-like kinase Cdc15, the polo kinase Cdc5, the LATS/NDR kinase Dbf2-Mob1, and the spindle pole body (SPB) scaffold protein Nud1 [13]. Regulation of MEN activators and inhibitors to ensure the coordinate and timely execution of mitotic exit is required for error-free cell division to occur. 

**Figure 1:**
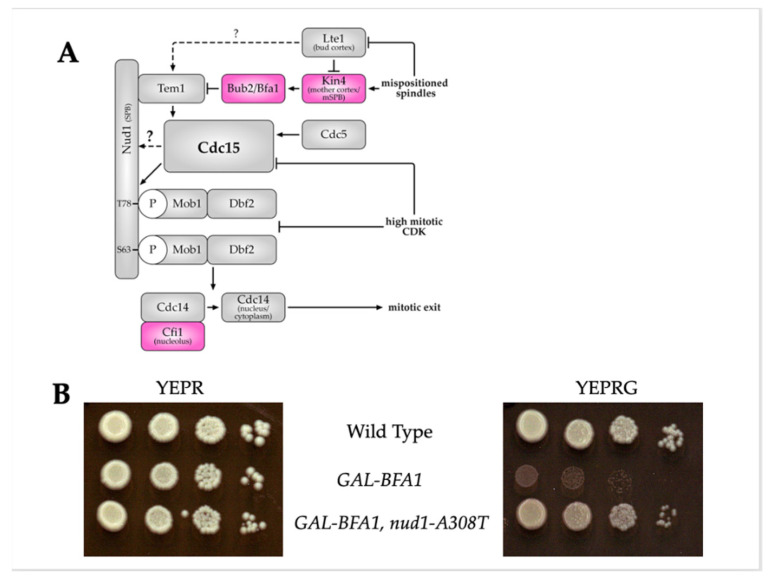
The *nud1-A308T* allele suppresses the growth defect of *GAL-BFA1* cells. (**A**) The mitotic exit network components and regulators are shown. Proteins that inhibit mitotic exit are depicted in red boxes, while activators are colored as grey boxes. The majority of the components assemble on the SPBs. The cellular locations of components that are not on this structure are indicated in parentheses underneath the protein names. See text for details. (**B**) Wild-type (AS3), *GAL-BFA1* (AS5), and *GAL-BFA1 nud1-A308T* (AS53) strains were grown in YEP + Raffinose (YEPR) overnight and 10-fold serial dilutions were spotted on YEPR (left) or YEPR + Galactose (YEPRG; right). The plates were incubated at 25 °C for 3 days.

The SPBs, which are yeast centrosome equivalents, act as a hub for the assembly of activated MEN components (see Figure 1A). The small Ras-like GTPase Tem1 functions at the top of the MEN pathway and provides an essential link between nuclear position and Cdc14 activation. Tem1 first accumulates on SPBs during anaphase upon mitotic spindle elongation, and preferentially associates with the daughter cell-bound SPB (dSPB) [14,15,16,17]. Prior to spindle elongation, the localization of Tem1 to the SPBs is countered by the MEN inhibitor Kin4, which is localized to the mother cell cortex and mother SPB (mSPB) [18,19]. Kin4 activates the two-component GTPase Bub2-Bfa1, which inhibits Tem1 if the SAC or the SPoC are triggered [20,21,22,23]. Movement of the dSPB into the bud compartment during anaphase relieves the inhibition on Tem1 localization to this organelle due to multiple functions of the MEN activator Lte1. Lte1 is localized to the bud cortex and antagonizes Kin4 in this cellular compartment [14,15,24,25,26]. This in turn allows Tem1 to associate with the dSPB, likely by binding to Bfa1 and Nud1 on the outer plaque of the dSPB [15,27,28]. Lte1 likely has additional unknown Tem1-activating functions [26]. Importantly, the spatial segregation of the Tem1-inhibitor Kin4 in the mother cell compartment, and the Tem1-activator Lte1 in the daughter cell compartment creates an MEN-inhibitory and MEN-activating zone, respectively, and promotes the coupling of nuclear migration with mitotic exit [29,30]. Prior work has shown that tethering Tem1 to both SPBs by creating fusions with SPB outer plaque components such as *SPC72* or *CNM67* or to the dSPB by tethering with *BFA1* leads to SPoC bypass and can accelerate anaphase [28,31]. On the other hand, removal of Tem1 from SPBs by the attachment of a CAAX plasma membrane-targeting signal or by overexpression of its SPB receptor *BFA1* can inhibit MEN activation [28,32]. Therefore, restriction of Tem1′s SPB association to anaphase cells with correct spindle position is essential for cell cycle control and ploidy maintenance (Figure 1A). 

Immediately downstream of Tem1 in the MEN pathway is the Hippo/PAK-like kinase Cdc15 [33,34,35]. Tem1 and the polo kinase Cdc5 act coordinately to recruit Cdc15 to SPBs where it becomes concentrated first at the dSPB and subsequently at the mSPB [17,36]. Cdc15 then phosphorylates Nud1 on the outer plaque of the SPBs at S63 and T78, creating SPB phospho-docking sites for Dbf2-Mob1 where it can be phosphorylated and activated by Cdc15 [37,38]. The Dbf2-Mob1 complex associates first with the mSPB, and subsequently with the dSBP [17]. The active Dbf2-Mob1 complex then translocates into the nucleolus to participate directly in the activation of Cdc14 by liberating the protein from its nucleolar inhibitor, Cfi1/Net1, and allowing the protein phosphatase to reach its targets in the nucleus and cytoplasm [39,40,41]. In sum, the inactivation of mitotic CDK activity and resulting mitotic exit is achieved by (1) the release of Cdc14 from the nucleolus that leads to reversal of the phosphorylation imposed by CDK, (2) mitotic cyclin degradation, and (3) the accumulation of the mitotic CDK-inhibitor Sic1. 

Just as Tem1 regulation provides spatial coordination for MEN activation, the regulation of the protein kinases Cdc5, Cdc15, and Dbf2-Mob1 is important for temporal coordination. Cdc5 kinase is activated, and the protein localizes to SPBs from S phase through mitosis, which poses some temporal restrictions on MEN activation [23,42,43,44]. However, the MEN remains inactive with Cdc14 sequestered in the nucleolus even when the SPBs makes brief forays into the bud during cells undergoing a prolonged mitotic metaphase arrest [45]. This is because the activity and SPB localization of both Cdc15 and Dbf2-Mob1 is inhibited by high mitotic CDK activity [45,46,47]. Therefore, MEN activity is restricted to anaphase (Figure 1A). It is only during this stage of mitosis where both a pool of mitotic CDKs is degraded following the metaphase–anaphase transition and a Tem1-bearing SPB enters the bud compartment that MEN can be activated [14,15,45,48]. When Cdc15 is forced to localize to SPBs outside of anaphase, premature MEN activity occurs, bypassing spatial and cell cycle progression checkpoints. For example, the tethering of Cdc15 to SPBs by fusing Cdc15 to the SPB component *CNM67* can lead to premature Dbf2-Mob1 kinase activity [36]. A *CDC15* allele, Cdc15(1-750), in which the resulting mutant protein is lacking its inhibitory C-terminus, similarly leads to SPB localization regardless of cell cycle stage and premature Dbf2-Mob1 kinase activity [36,49]. Both hyperactivated alleles of *CDC15* circumvent the requirement for the spatial sensor Tem1 and the cell cycle clock sensor Cdc5 to generate an active pool of Cdc15 [36]. Thus, restricting Cdc15 SPB localization to anaphase is necessary to coordinate MEN activation with spatial and temporal signals. Despite its importance, the question of how Cdc15 associates with SPBs is not yet well understood. 

In this study, we have identified and characterized an allele of the MEN scaffold *NUD1*, *nud1-A308T*, as a spontaneous suppressor of *GAL-BFA1* lethality. This allele exhibits a partial bypass of the SAC and a robust bypass of the SPoC. In addition, we show that cells containing *nud1-A308T* exhibit a shortened anaphase length. Our data suggest that the A308T mutation in *NUD1* results in a hyperactive scaffold, causing the recruitment of the MEN kinase Cdc15 in all phases of the cell cycle, and the early recruitment of Dbf2-Mob1 in metaphase cells. Furthermore, *nud1-A308T* cells display a reduced requirement for the polo kinase Cdc5 in MEN activation. Our data highlight the critical importance of scaffold regulation in preserving the integrity of scaffold-assisted signaling pathways. 

## 2. Materials and Methods

### 2.1. Yeast Strains and Growth Conditions

All yeast strains used in this study were derivatives of W303 (AS3). The relevant genotypes of the strains used in this study were indicated in Table 1 below. The *GAL-nud1-T78A*, *GAL-nud1-S53A*, *S63A*, *T78A*, and *GAL-mob1-77* constructs were described in [38]. Culturing conditions were described in the figure legends. 

### 2.2. Fixed-Cell Imaging and Analysis

Indirect immunofluorescence was performed as described previously for anti-tubulin (YOL1/34, Abcam) [50]. Images for Supplementary Figure S1 were acquired using a Zeiss Axioplan 2 (Zeiss, Thornwood, NY, USA) with a Hamamatsu Orca-R2 camera (Hamamatsu, Middlesex, NJ, USA) and a 63× objective. Cells for Figures 3 and 5 were fixed in a 4% paraformaldehyde, 3.4% sucrose solution for 15 min. Cells were washed once in potassium phosphate/sorbitol buffer (1.2 M sorbitol, 0.1 M potassium phosphate, pH 7.5), and treated with 1% Triton X-100 for 5 min. Cells were washed again in potassium phosphate/sorbitol buffer and resuspended in potassium phosphate/sorbitol buffer containing 4′, 6-diamidino-2-phenylindole dihydrochloride before imaging. These cells were imaged using a DeltaVision Elite microscope (GE Healthcare Bio-Sciences, Pittsburgh, PA, USA) with an InsightSSI solid-state light source, a CoolSNAP HQ2 camera, and a 60× plan-ApoN objective. Cells for budding and re-budding analyses were fixed in 3.7% formaldehyde, 0.1 M potassium phosphate, pH 6.4 prior to brief sonication and analysis. 

### 2.3. Live-Cell Imaging and Analysis

Live cell movies for Figures 4C,D and 7C were done on agarose pads (2% agarose, synthetic complete (SC) medium containing 2% glucose. Cells for Figures 6 and 7A,B were imaged directly from log phase cultures. These cells were imaged using a DeltaVision Elite microscope (GE Healthcare Bio-Sciences, Pittsburgh, PA, USA) with an InsightSSI solid-state light source, an UltimateFocus hardware autofocus system, and a model IX-71 Olympus microscope (Center Valley, PA, USA) controlled by SoftWoRx software. A 60× Plan APO 1.42NA objective and CoolSNAP HQ2 camera (Teledyne Photometrics, Tuscon, AZ, USA) were used for image acquisition. Fiji software was used for image processing and analysis. To quantify anaphase length, image processing was performed by Volocity (PerkinElmer, Waltham, MA, USA) software.

### 2.4. Protein Extraction, Immunoprecipitation, and Western Blot Analysis

Approximately 25ODs of log phase cells grown in indicated conditions (see Figure 9) were pelleted and cell pellets were resuspended in cold NP40 buffer, containing 2X HALT Protease Inhibitor Cocktail (Promega, Madison, WI, USA), 60 mM β-glycerophosphate, 15 mM PNPP, 1 mM DTT, and 100 μM sodium orthovanadate. Cells were lysed by bead-beating using a chilled MiniBeadbeater (BioSpec Products, Bartlesville, OK, USA; 4 rounds of 60 s each) and glass beads. Protein extracts were cleared using a microcentrifuge and the protein concentration in each lysate was determined by Bradford assay (Bio-Rad, Hercules, CA, USA). Equilibrated anti-HA agarose beads (Sigma-Aldrich, St. Louis, MO, USA) were used to immunoprecipitate Mob1-6HA for 2 h at 4 °C. The reactions were washed with NP40 buffer six times before boiling in SDS PAGE protein buffer (Bio-Rad) for 5 min. Proteins were resolved on a 4–20% gradient gel (Bio-Rad) prior to transfer onto a nitrocellulose membrane. Mob1-6HA was detected using mouse anti-HA (Invitrogen, Waltham, MA, USA) at a 1:1000 dilution and Nud1-3V5 was detected using mouse anti-V5 (Invitrogen) at a 1:2000 dilution. Secondary antibodies were at 1:2000 and 1:5000, respectively. Blots were imaged using the ECL Plus system (GE Healthcare Bio-sciences).

## 3. Results

### 3.1. Nud1-A308T Partially Suppresses the Cell Cycle Defects of GAL-BFA1

Tem1 localization to the spindle pole body is highly dynamic and is regulated by the two component GTPase activating protein Bfa1-Bub2 [15,16,17,28,31]. It was previously shown that the overexpression of *BFA1* using a galactose-inducible promoter caused inhibition of mitotic exit in a *BUB2*-independent manner [51]. We demonstrated that this defect is due to the role of Bfa1 in Tem1-localization to the SPB and that the defect could be suppressed by concomitant overexpression of *TEM1* [32]. We sought to uncover new modes of MEN regulation by selecting for and characterizing spontaneous suppressors of *GAL-BFA1* lethality. Several of the spontaneous suppressors that we obtained by plating *GAL-BFA1* cells on galactose-containing plates caused decreased expression of *GAL-BFA1.* However, one suppressor strain did not (see Supplementary Figure S1). We sequenced the genome of this strain and identified a point mutation in the *NUD1* gene locus that changed the alanine 308 residue into a threonine (*nud1-A308T*). As seen in Figure 1B, when cells containing *GAL-BFA1* were plated on galactose containing media, this caused a severe growth defect that was largely suppressed by the presence of *nud1-A308T*.

### 3.2. Nud1-A308T Exhibits Weak Bypass of the Spindle Assembly Checkpoint

Upon treatment of cells with a microtubule poison, such as nocodazole, the SAC becomes activated and results in indefinite arrest in metaphase. This arrest is maintained by inhibition of mitotic exit. It has been previously shown that hyper-activation of the MEN, such as by deletion of the inhibitor *BUB2*, results in bypass of the SAC-induced metaphase arrest, leading to inappropriate exit from mitosis and re-budding [22]. The SAC can also be bypassed using a hyperactive version of an MEN activator. For example, overexpression of *TEM1* through a galactose promoter has been shown to bypass the SAC resulting in re-budding [52]. We suspected that this novel allele of *NUD1* caused a hyper-activated MEN phenotype, and we were interested in determining the level of hyperactivity exhibited by this allele. We decided to test the ability of *nud1-A308T* to bypass the SAC in the metaphase.

**Figure 2:**
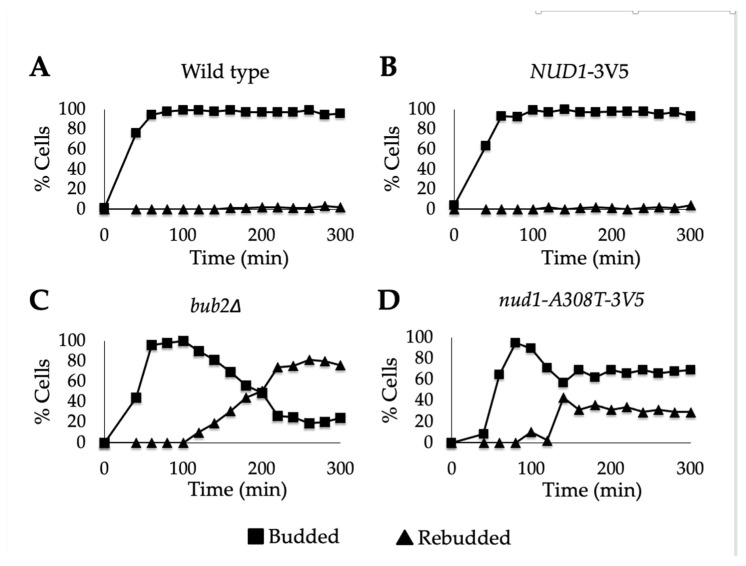
*nud1-A308T* partially bypasses the spindle assembly checkpoint (SAC). Log phase wild-type (AS3), *NUD1-3V5* (AS326), *bub2Δ* (AS112), and *nud1-A308T* (AS387) cells were arrested in the presence of alpha-factor pheromone (5 ug/mL) for a total of 3 h at 25 °C. Cells were then released into medium containing nocodazole (15 ug/mL) and samples were taken at the indicated times for budding analysis. After 120 min and 240 min, 7.5 ug/mL nocodazole was re-added to maintain the metaphase block. The percentage of budded (squares) and re-budded (triangles) cells observed at each time point in wild-type (**A**), *NUD1-3V5* (**B**), *bub2Δ* (**C**), and *nud1-A308T-3V5* (**D**) cells was plotted (*n* = 100).

Alpha factor arrested G1 cells were synchronously released into nocodazole-containing media. Samples were taken approximately every 30 min and analyzed for re-budding. The percentage of budded and re-budded cells was graphed. As expected, almost no re-budding was observed in the wild-type and *NUD1-3V5* cells (Figure 2A,B). In *bub2∆* cells, where SAC is bypassed, re-budded cells appeared beginning at 120 min and plateaued at around 80% (Figure 2C). In *nud1-A308T* cells, an increase in re-budded cells occurred at 140 min, but re-budding plateaued at around 30%. Therefore, we concluded that *nud1-A308T* caused a weak bypass of the SAC, unlike cells that completely lack the MEN inhibitor *BUB2*.

### 3.3. Nud1-A308T Suppresses the Spindle Position Checkpoint

**Figure 3:**
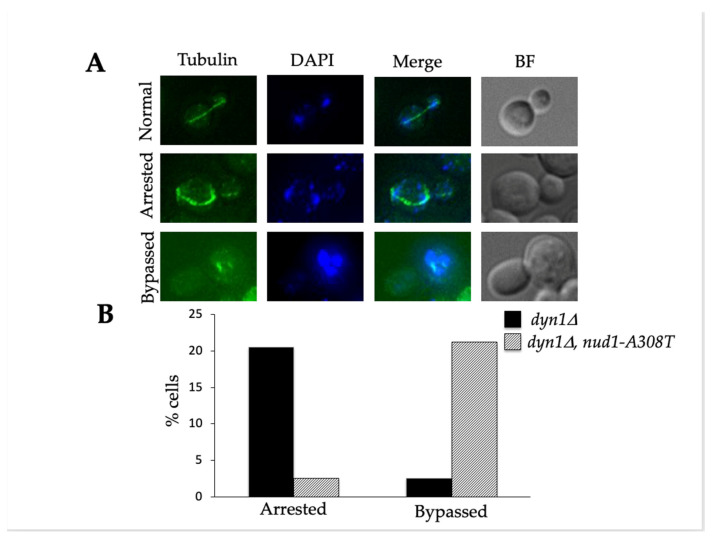
The *nud1-A308T* allele causes bypass of the Spindle Position Checkpoint (SPoC). Log phase wild-type (AS417) and *nud1-A308T* (AS474) cells containing both *dyn1Δ* and *GFP-TUB1* were grown at 14 °C for 25 h to induce spindle mispositioning. Samples were collected and the cells were fixed with paraformaldehyde and stained with DAPI prior to imaging. (**A**) Representative images of a cell undergoing a proper anaphase (normal), arrested in anaphase with a mispositioned spindle (arrested), and with multiple nuclei and spindles indicating SPoC bypass (bypassed) are shown. (**B**) The percentage of cells from each strain with arrested or bypassed morphology was plotted (*n* > 500).

In addition to looking at the ability of Nud1-A308T to bypass the SAC, we also looked at the ability of Nud1-A308T to bypass SPoC. It was previously shown that hyperactive MEN components, such as *TEM1-eGFP*, harbor the ability to bypass the SPoC and complete mitotic exit despite misaligned spindles [52]. Spindle mispositioning can be induced by growing cells lacking the *DYN1* spindle positioning motor protein at low temperatures [11]. Wild-type and *nud1-A308T* cells containing a deletion of the *DYN1* locus were cultured at 14 °C to induce spindle mispositioning. Samples were taken after 24 h and imaged. As expected, we observed cells with properly aligned spindles going through anaphase and cells with misaligned spindles arresting in anaphase. We also saw a third category of cells with multiple nuclei. This is indictive of cells with misaligned spindles inappropriately exiting from mitosis and therefore bypassing the SPoC (Figure 3A). The percentage of cells from wild-type and *nud1-A308T* strains with arrested or bypassed morphology was plotted. In wild-type cells, 20% of cells were arrested in anaphase, while approximately 2.5% of cells bypassed the SPoC, resulting in multiple nuclei (Figure 3B). In *nud1-A308T* cells, 2.5% of cells were arrested in anaphase, while 20% of cells bypassed the SPoC (Figure 3B). We concluded that *nud1-A308T* exhibits a robust bypass of the SPoC. 

### 3.4. Nud1-A308T Cells Have a Shortened Anaphase

**Figure 4:**
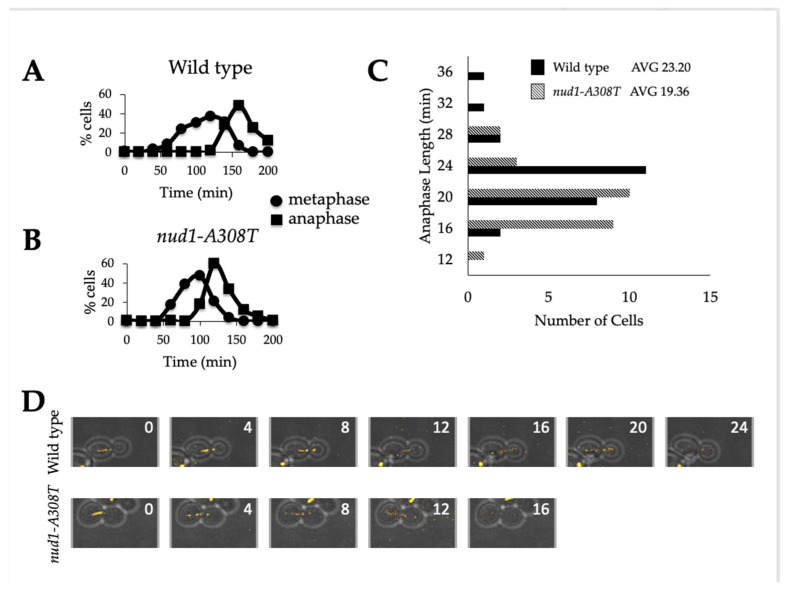
Mutants containing *nud1-A308T* have a shortened anaphase duration. (**A**) and (**B**): wild-type (AS138) and *nud1-A308T* (AS303) cells were grown to log phase and arrested in 5 ug/mL alpha-factor pheromone for 2.5 h. The cells were then released into medium lacking pheromone and samples were taken at the indicated times for tubulin immunofluorescence. The percentage of cells with metaphase (circles) or anaphase (squares) morphology in wild-type (**A**) and *nud1-A308T* (**B**) strains was quantified and plotted (*n* = 100). (**C**) and (**D**): Wild-type (AS476) and *nud1-A308T* (AS477) cells harboring Tub1-mCherry and Dbf2-eGFP fusions were grown to log phase in SC + glucose medium and living cells growing on an agar pad were imaged every 4 min for a total of 4 h. (**C**) The start and completion of anaphase was marked and the number of cells with anaphase duration of 16, 20, 24, or 28 min in each strain was plotted (*n* > 19). The average duration of anaphase in each strain is indicated above the graph. (**D**) A representative wild-type and *nud1-A308T* cell is shown.

Previous studies demonstrated that hyperactive alleles of MEN mutants exhibited decreased anaphase length [17]. Therefore, we wanted to analyze the length of anaphase in individual cells harboring *nud1-A308T*. Both wild-type and *nud1-A308T* cells were synchronously released from a G1 alpha factor arrest into media lacking pheromone. Samples were taken at indicated time points and processed for tubulin immunofluorescence in order to visualize spindle morphology. Anaphase lasted for approximately 70 min in wild-type cells (Figure 4A) but for 60 min in *nud1-A308T* cells (Figure 4B). In order to more precisely quantify this difference in anaphase length, we performed live cell imaging of wild-type and *nud1-A308T* cells proceeding through the cell cycle and analyzed the length of anaphase in individual cells. Anaphase onset was determined by a spindle length of 3 μm or longer and anaphase completion was determined by spindle breakdown (Figure 4D). We quantified the length of anaphase in 19 or more cells from each genotype and observed that wild-type cells have an average anaphase length of 23.3 min, while *nud1-A308T* cells have an average anaphase length of 19.36 min (Figure 4C). These results indicated that the presence of the Nud1-A308T mutant protein has an effect on anaphase progression. This could be due to cells having the ability to pass through the metaphase to anaphase transition without satisfying the checkpoint, as is suggested by the data in Figure 2. 

### 3.5. Nud1-A308T Does Not Promote Association of Tem1 to SPBs

If *nud1-A308T* cells progress from metaphase to anaphase without satisfying the SAC, as our data indicate, this could be caused by early activation and recruitment of an MEN component by the mutant Nud1-A308T scaffold. One possible mechanism of action would be early or increased recruitment of Tem1 to the SPBs, as this has been shown to hyperactivate the MEN [17,29,31]. The *nud1-A308T* allele was discovered as a suppressor of lethality in *GAL-BFA1* cells, in which Tem1 localization is disrupted (Figure 1B; [32]). Therefore, we looked at Tem1 localization in *GAL-BFA1 nud1-A308T* cells to determine whether Tem1 recruitment was responsible for the suppression phenotype.

**Figure 5:**
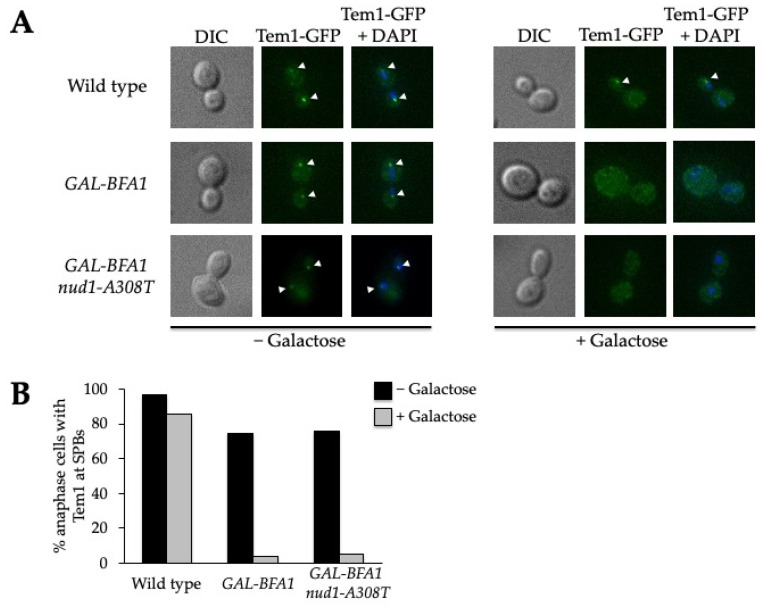
The *nud1-A308T* allele does not promote association of Tem1 to SPBs. Wild-type (AS15), *GAL-BFA1* (AS79), and *GAL-BFA1 nud1-A308T* (AS296) cells all harboring a Tem1-GFP fusion were grown to log phase at 30 °C in YEPR; 2% galactose was then added to induce the overexpression of *BFA1* for a total of 3 h. Samples were taken just prior to galactose induction and after 3 h induction and the cells were fixed in paraformaldehyde prior to imaging. (**A**) Representative composite images of anaphase cells for each strain prior to galactose addition, and following a 3 h galactose induction are shown. The DNA stained with DAPI is shown in blue and Tem1-GFP is in green. Arrowhead indicates Tem1-GFP localization to the SPB. (**B**) The percentage of anaphase cells with Tem1-GFP localized to one or both SPBs in the absence of galactose (black) and 3 h (gray) after galactose addition for each strain was plotted. (*n* > 100 cells).

We examined Tem1 localization by fixing samples of wild-type, *GAL-BFA1,* and *GAL-BFA1 nud1-A308T* cells containing Tem1-GFP before and after the induction of *BFA1* overexpression using galactose. The percentage of anaphase cells with Tem1 localized to one or both SPB before and after galactose addition was plotted for each strain (Figure 5A). In wild-type anaphase cells, Tem1 was localized to the SPBs as expected regardless of culture medium. In control *GAL-BFA1* cells, Tem1 only localized to SPBs when galactose was not present. Interestingly, cells containing *GAL-BFA1 nud1-A308T* appeared similar to *GAL-BFA1* alone, and Tem1 was not localized to SPBs during anaphase when *BFA1* was overexpressed (Figure 5). These data showed that the mutant Nud1-A308T does not function by restoring the Tem1 localization defect in cells overexpressing *BFA1*. This was surprising since Tem1 is known to bind to the Nud1 MEN scaffold [28,53].

### 3.6. Cdc15 Is Recruited to SPBs in All Cell Cycle Phases in the Majority of Nud1-A308T Cells

**Figure 6:**
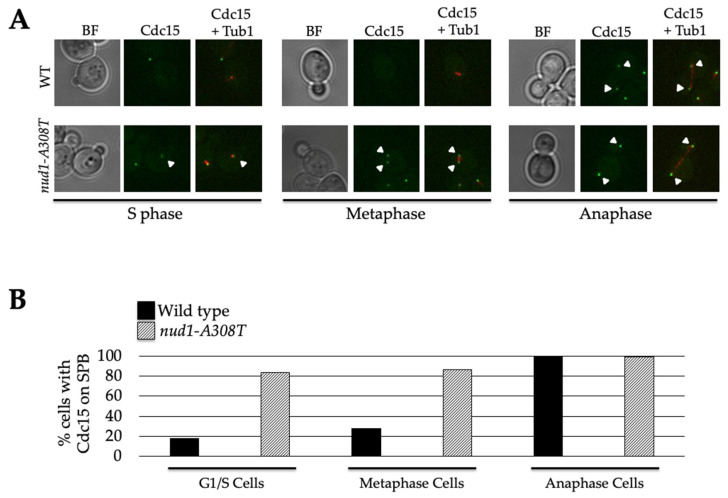
Cdc15 is recruited to SPBs in all cell cycle phases in *nud1-A308T* cells. Wild-type (AS513) and *nud1-A308T* (AS517) cells harboring Cdc15-eGFP and Tub1-mCherry fusions were grown to log phase in SC + glucose medium and living cells were imaged. (**A**) Brighfield (BF), Cdc15, and Cdc15 and Tub1 composite representative wild-type and *nud1-A308T* cells from S phase, metaphase, and anaphase are shown. Arrows indicate Cdc15 localization to SPBs. (**B**) The percentage of G1/S, metaphase, and anaphase cells containing Cdc15 on one or more SPBs in wild-type (black) or *nud1-A308T* (striped) cells is plotted (*n* ≥ 80 cells for each stage).

After concluding that Nud1-A308T was not acting by recruiting Tem1, we were interested in looking at Cdc15 SPB localization in *nud1-A308T* cells. Cdc15 is known to localize to SPBs upon its activation [33,34,35]. Cdc15 is also responsible for creating Nud1 phospho-docking sites to recruit and activate Dbf2-Mob1, but the mechanism of Cdc15 interaction with SPBs is not currently understood [38]. In order to analyze Cdc15 localization, we imaged wild-type and *nud1-A308T* cells harboring Cdc15-eGFP and Tub1-mCherry and quantified the percentage of G1/S, metaphase, and anaphase cells with Cdc15 localized to one or more SPB (Figure 6). Spindle morphology was used to determine which stage of the cell cycle cells were in. In wild-type cells, Cdc15 was localized in almost 100% of anaphase cells, while only 17% of G1/S cells and 27% of metaphase cells exhibited SPB localization (Figure 6B). In contrast, in the presence of *nud1-A308T* 84% of G1/S cells and 87% of metaphase cells exhibited Cdc15 SPB localization (Figure 6A,B). These data indicated that the *nud1-A308T* allele was hyperactive in MEN activation due to unscheduled recruitment of Cdc15 SPBs. 

### 3.7. Nud1-A308T Prematurely Recruits Dbf2 to the Mother SPB in Metaphase

**Figure 7:**
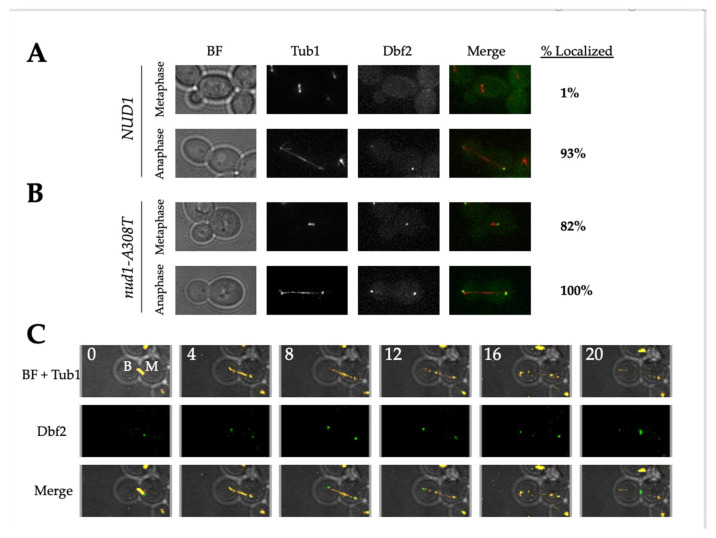
*nud1-A308T* recruits Dbf2 asymmetrically to the mSPB in metaphase. (**A**) and (**B**): wild-type (AS476) and *nud1-A308T* (AS477) cells harboring Dbf2-eGFP and Tub1-mCherry fusions were grown to log phase in SC + glucose media. Live cells were imaged. Representative metaphase and anaphase cells for each genotype are shown. The percentage of cells in each phase with Dbf2-eGFP localized to one or two SPBs is indicated in the column to the right of the images (*n* = 100). (**C**) *nud1-A308T* (AS477) cells harboring Dbf2-eGFP and Tub1-mCherry fusions were grown on an SC + glucose agar pad and imaged every 4 min for a total of 4 h. The start of metaphase was determined by identifying spindles that were 2 microns in length and this time was marked as 0 (*n* = 20). A representative cell is shown. The bud compartment is labeled “B” and the mother compartment is labeled “M”.

It had previously been shown that cells with premature Cdc15 SPB localization also resulted in premature association of Dbf2-Mob1 to the SPBs [36,38]. After seeing the increase in Cdc15 localization, we wanted to determine whether Dbf2 localized to the SPB earlier in *nud1-A308T* cells. To carry this out, we imaged wild-type and *nud1-A308T* cells harboring Dbf2-eGFP and Tub1-mCherry. We then quantified the percentage of cells in metaphase and anaphase with Dbf2 localized to one or two SPBs (Figure 7). In wild-type cells, 1% of metaphase cells had Dbf2 localized to the SPB and 93% of anaphase cells had Dbf2 localized to the SPB (Figure 7A). In *nud1-A308T* cells, 82% of metaphase cells had Dbf2 localized to one SPB and 100% of anaphase cells had Dbf2 localized to both SPBs (Figure 7B). We wanted to identify whether Dbf2 localized to the mSPB or the dSPB during metaphase in *nud1-A308T* cells. To do this we performed live cell imaging of *nud1-A308T* cells growing on agar pads. Images were taken every 4 min and spindle morphology and Dbf2 localization were analyzed. In 100% of metaphase cells analyzed (*n* = 20), *nud1-A308T* cells had Dbf2 localized to the mSPB only (Figure 7C). These results indicated that the mutant Nud1-A308T caused premature recruitment of Dbf2 to the mSPB during metaphase. 

### 3.8. Nud1-A308T Function Requires CDC15 and MOB1, but Not CDC5

Now that we had established that Cdc15 was recruited in all cell cycle phases, and that this resulted in early recruitment of Dbf2-Mob1 in metaphase, we wanted to determine whether *CDC15, MOB1*, and *CDC5* were required for the MEN activating function of *nud1-A308T*. As described previously, the localization of Cdc15 to SPBs in wild-type cells requires the activity of both Tem1 and Cdc5 [36]. We had already shown that the Nud1-A308T mutant was not acting by recruiting Tem1 (Figure 5), but we wondered whether this mutant scaffold could also bypass the requirement for Cdc5 activity in Cdc15 recruitment. On the other hand, we expected that if the mechanism of action of *nud1-A308T* was through Cdc15, then both *CDC15* and *MOB1* would be required for MEN activation in this mutant. 

**Figure 8:**
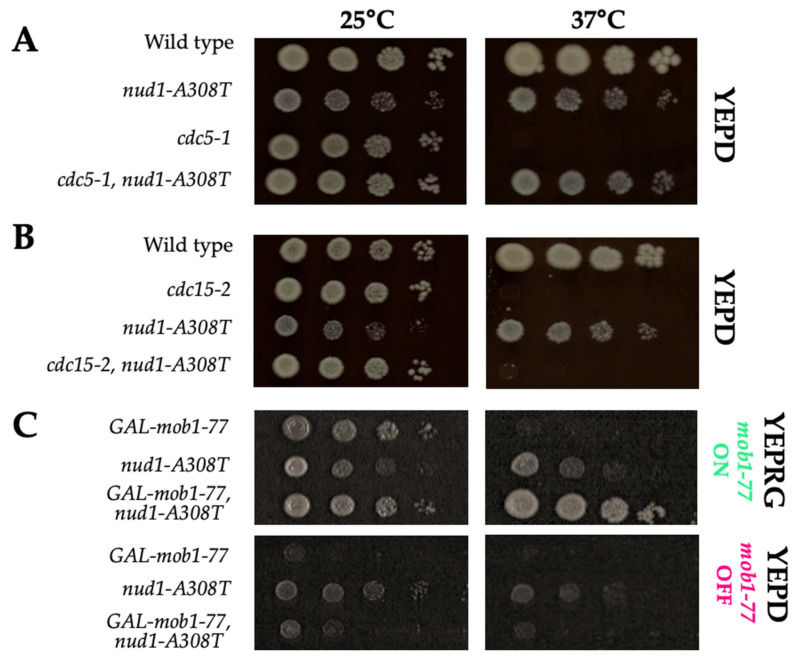
Nud1-A308T function requires *CDC15* and *MOB1* but not *CDC5*. (**A**) Ten-fold dilutions of cells containing *NUD1* (AS3), *nud1-A308T* (AS387), *cdc5-1* (AS564), and *nud1-A308T, cdc5-1* (AS570) were spotted onto YEPD plates that were incubated at 25 °C or 37 °C for 2 days before imaging. (**B**) Ten-fold dilutions of cells containing *NUD1* (AS3), *cdc15-2* (AS179), *nud1-A308T* (AS387), or *nud1-A308T*, *cdc15-2* (AS447) were spotted onto YEPD plates and incubated at 25 °C or 37 °C for 2–3 days before imaging. (**C**) Ten-fold dilutions of cells containing *GAL-mob1-77* (AS402), *nud1-A308T* (AS387), or both *GAL-mob1-77* and *nud1A308T* (AS478) were spotted onto YEPRG (top row) or YEPD (bottom row) plates to activate or to repress the expression of *mob1-77*. The plates were incubated at 25 °C or 37 °C for 2 days before imaging.

To determine if *CDC5*, *CDC15*, and *MOB1* were required for *nud1-A308T*, we crossed the *nud1-A308T* allele into cells containing temperature-sensitive mutations in each of these MEN components. When cells that were double mutant for the kinase-defective *cdc5-1* allele and *nud1-A308T* were grown at the restrictive temperature of 37 °C, we were surprised to find that they were able to grow almost as well as wild-type (Figure 8A). These data demonstrated that *nud1-A308T* recruits Cdc15 to SPBs independently of both *TEM1* and *CDC5*. As expected, when *cdc15-2 nud1-A308T* double mutants were grown at the restrictive temperature of 37 °C, the cells resembled the phenotype of *cdc15-2* alone (Figure 8B). This result confirmed that the mechanism by which the Nud1-A308T mutant scaffold is acting involves Cdc15 recruitment. 

To confirm that Mob1, which acts downstream of Cdc15, was also required for MEN activation in the *nud1-A308T* background, we combined this mutation with the *GAL-mob1-77* allele. In the W303 strain background, the *mob1-77* allele causes a less severe growth defect at 37 °C than in the S288C strain background, where this allele was originally characterized [38,54]. Therefore, we examined the double mutant in cells where the *mob1-77* allele was placed under the *GAL*-inducible promoter to allow for complete depletion of *MOB1* by growth in medium containing glucose [38]. In the presence of galactose at 25 °C, all strains were able to grow. However, at 37 °C, when galactose was present, cells with *GAL-mob1-77* were unable to grow while the presence of *nud1-A308T* suppressed this growth defect (Figure 8C, top YEPRG panels). In the presence of glucose, where the expression of *GAL-mob1-77* was repressed, the *nud1-A308T* allele was unable to suppress the growth defect at either temperature (Figure 8C, bottom YEPD panels). These results demonstrated that the presence of Mob1 was also required for Nud1-A308T to activate the MEN. However, interestingly, the mutant scaffold was able to suppress the MEN defect of W303 cells containing *mob1-77* when the protein was overexpressed.

### 3.9. Nud1-A308T Recruits Mob1 in Metaphase and Suppresses the Dominant GAL-nud1-T78A

**Figure 9:**
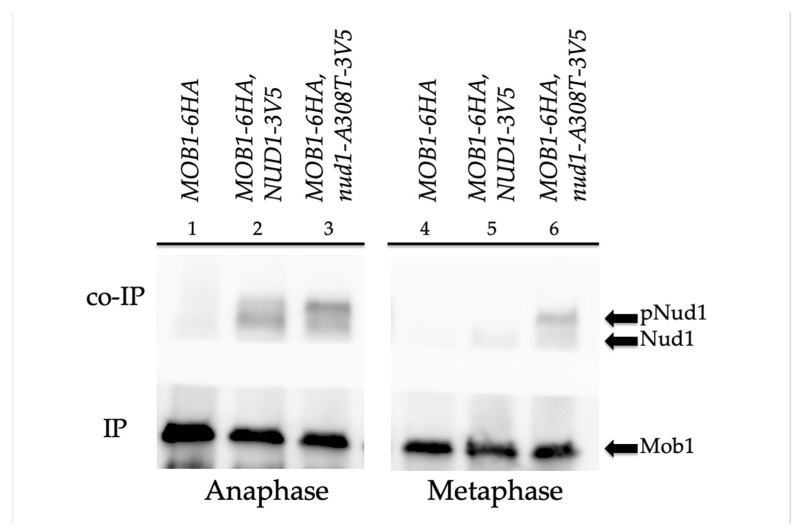
The mutant Nud1-A308T protein recruits Mob1 in both metaphase and anaphase cells. *MOB1-6HA* (AS407), *MOB1-6HA NUD1-3V5* (AS228), and *MOB1-6HA nud1-A308T-V5* (AS439) cells harboring the *cdc14-3* mutation were grown to log phase. Cell cultures were split, and half of each culture was incubated at 37 °C for two hours to inactivate Cdc14 and produce an anaphase arrest (samples 1–3). The other half of each culture was arrested with 15 μg/mL nocodazole at 25 °C to produce a metaphase arrest (samples 4–6). Protein extracts were produced from each sample and Mob1-6HA was immunoprecipitated using anti-HA agarose beads. The samples were analyzed by SDS-PAGE and Western blotting using anti-HA antibody to detect the amount of Mob1-6HA immunoprecipitated in each sample and anti-V5 antibodies to detect the amount of Nud1-3V5 or Nud1-A308T-3V5 co-immunoprecipitated. Mob1, Nud1 (samples 2 and 5), and Nud1-A308T (samples 3 and 6) are labeled on the blot. Higher migrating phosphorylated species of Nud1 and Nud1-A308T are labeled (pNud1).

Taken together, our data suggested a likely model where the mutant Nud1-A308T scaffold led to hyperactivation of the MEN by recruitment of Cdc15 and a resulting early phosphorylation of Nud1 phospho-docking sites; whereby, Dbf2-Mob1 could then be recruited and activated. We decided to further test this by examining whether the Nud1-A308T mutant protein could associate with Mob1 in metaphase by co-immunoprecipitation analysis. It had been previously demonstrated that higher migrating forms of Nud1 corresponding to phosphorylation accumulated in anaphase and that Nud1 could associate with Mob1 specifically in anaphase [38]. We arrested *MOB1-6HA* cells containing Nud1 tagged with a 3V5 epitope or the mutant Nud1-A308T-3V5 protein in metaphase using nocodazole or in anaphase using the *cdc14-3* temperature-sensitive allele. Upon immunoprecipitating Mob1-6HA, we observed that both Nud1-3V5 and Nud1-A308T-3V5 were pulled down in cells arrested in anaphase (Figure 9, left panel). In contrast, only Nud1-A308T-3V5 could associate with immunoprecipitated Mob1-6HA in metaphase-arrested cells, while the wild-type Nud1 protein did not associate (Figure 9, right panel). Interestingly, higher migrating forms of Nud1-A308T were associated with Mob1 in both metaphase and anaphase-arrested cells. Therefore, we next decided to explore the role of Nud1 phosphorylation on Nud1-A308T function more directly. 

**Figure 10:**
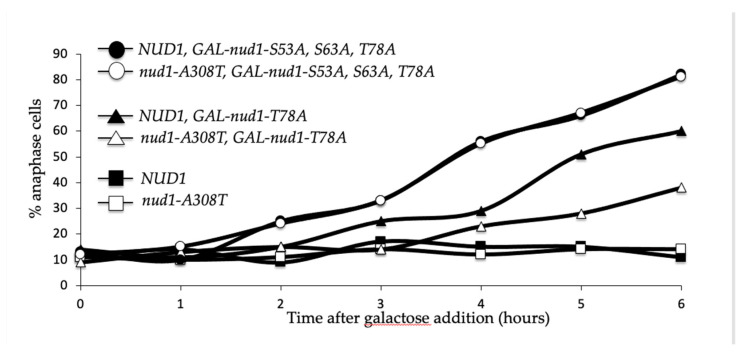
*nud1-A308T* suppresses the dominant GAL-nud1-T78A. *NUD1* (AS326), *nud1-A308T* (AS387), *NUD1 GAL-nud1-T78A* (AS384), *nud1-A308T GAL-nud1-T78A* (AS395), *NUD1 GAL-nud1-S53A*, *S63A*, *T78A* (AS388), and *nud1-A308T GAL-nud1-S53A, S63A, T78A* (AS390) cells were grown to log phase in YEPR and subsequently induced with 2% galactose addition. Samples were taken at the indicated times and processed for tubulin immunofluorescence. The percentage of cells with anaphase spindles at each time point in each strain was determined and plotted (*n* = 100–200).

Normally, when Cdc15 interacts with SPBs, three important Nud1 residues are phosphorylated. These sites are serine 53, serine 63, and threonine 78 (hereafter S53, S63, and T78). Only S63 and T78 are Cdc15-dependent, and T78 is the phosphorylation site most vital for MEN activation [38]. We were interested in determining whether *nud1-A308T* required the presence of these known phosphorylation sites. To do this we introduced *nud1-A308T* into a strain with the *GAL-nud1-S53A,S63A,T78A* allele integrated at the *TRP1* locus. This is a version of Nud1 with all three key phosphorylation sites muted to alanine, which prevents the formation of the Dbf2-Mob1 phospho-docking sites [38]. This allele is under the *GAL*-promoter allowing overexpression of Nud1 with nonfunctional phosphorylation sites in the presence of galactose. We also utilized the *GAL-nud1-T78A* allele, in which only site T78 is mutate to alanine [38]. As expected, when grown for several hours in the presence of galactose 80% of *GAL-nud1-S53A, S63A* and *T78A* cells, also containing wild-type *NUD1*, were arrested in anaphase. The *GAL-nud1-T78A* allele containing wild-type *NUD1* had a less severe anaphase arrest phenotype as previously demonstrated ([38]; Figure 10, closed circles and closed triangles). In cells containing both *GAL-nud1-S53A,S63A,T78A*, and *nud1-A308T*, there was no suppression of the dominant anaphase arrest phenotype (Figure 10, open circles). Intriguingly, while 55% of *GAL-nud1-T78 NUD1* cells arrested in anaphase after 6 h growth in galactose-containing medium, only 30% of *GAL-nud1-T78A nud1-A308T* double mutant cells arrested (Figure 10, triangles). Taken together, these data indicated that at least one of the phosphorylation sites was needed after Cdc15 recruitment to allow for MEN activation when the mutant scaffold was present. However, the increased Cdc15 recruitment to SPBs as a result of the mutant Nud1-A308T protein could suppress the defects associated with overexpression of *NUD1* containing a non-functional T78 phosphorylation site. 

## 4. Discussion

The MEN is essential in budding yeast to execute the exit from mitosis transition during the cell division cycle. Proper regulation of MEN components is necessary to ensure that exit from mitosis is coordinated with both cell cycle clock progression and migration of the daughter cell nucleus into the bud compartment. In this study, using a spontaneous genetic suppressor screen of *GAL-BFA1* lethality, we have identified a hyperactive allele of the MEN scaffold *NUD1*. The *nud1-A308T* allele exhibited bypass of the SPoC in anaphase cells with mispositioned spindles, resulting in the accumulation of anucleate and multinucleate cells (Figure 3). In addition, *nud1-A308T* exhibited a mild bypass of the SAC in metaphase-arrested cells (Figure 2), demonstrating that proper regulation of Nud1 is important to maintain genome integrity when mitotic checkpoints are activated. 

The *nud1-A308T* allele could suppress the mitotic exit defects of cells impaired for Cdc5 and Dbf2-Mob1 kinase activity. However, cells that were impaired for Cdc15 activity and cells completely lacking *MOB1* expression were not rescued by the mutant Nud1 scaffold (Figure 8). These data are consistent with the model that the *nud1-A308T* allele is hyperactive due to unscheduled recruitment of Cdc15 and resulting phosphorylation of the scaffold at S63 and T78, which prematurely creates Dbf2-Mob1 phospho-docking sites. Several lines of evidence support this model. The hyperactive Nud1 scaffold resulted in localization of Cdc15 to the SPB in all phases of the cell cycle, and therefore led to premature SPB recruitment and activation of the downstream Dbf2-Mob1 kinase in metaphase (Figure 6 and Figure 7). In addition, in cells containing a single-copy of *nud1-A308T*, the dominant negative effects of overexpressing non-phosphorylatable Nud1 mutants, such as *GAL-nud1-T78A*, *GAL-nud1-S53A*, *T78A*, and *GAL-nud1-S63A*, *T78A* are partially suppressed ([38]; Figure 10, Supplementary Figure S1). Lastly, Mob1 is associated with higher migrating forms of Nud1-A308T in both metaphase- and anaphase-arrested cells by co-immunoprecipitation analysis (Figure 9). 

It is notable that the presence of *nud1-A308T* causes cells to grow more slowly at both 25 °C and 37 °C. However, with the presence of temperature-sensitive mutations in MEN components, such as *cdc5-1* and *mob1-77*, the growth defects at 37 °C were ameliorated (see Figure 8A,C). These defects were likely due to the premature localization of Cdc15 to SPBs in the mutant, which led to MEN hyperactivation. The expression of the CDC15-SPB allele, in which *CDC15* was fused to the SPB outer plaque component *CNM67*, was found to be lethal unless the allele was placed under the control of the low-strength *MET3* promoter, indicating that cells cannot tolerate the constitutive localization of Cdc15 to SPBs well [36]. An additional non-mutually exclusive possibility for the poor growth of the *nud1-A308T* mutant could be that there is increased spindle mispositioning in this mutant. Nud1 is important for astral microtubule organization and spindle position, as shown by defects in both these processes that become apparent in the presence of the *nud1-44* allele and the *nud1-42A* allele. This is due to the role of Nud1 in localization of the astral microtubule anchor Spc72 [23,38,53]. We noticed the slightly increased presence of mispositioned spindles in all strains with the *nud1-A308T* allele, indicating that this could be another reason for decreased viability (AS unpublished observations). 

The *nud1-A308T* allele causes an interesting Dbf2-Mob1 phenotype—the asymmetric recruitment of the complex to the mSPB in metaphase cells (Figure 7C). Rock et al. demonstrated that expression of the *CDC15-SPB* allele also led to early recruitment of Mob1, in that case, in all phases of the cell cycle [38]. Therefore, we conclude that the *nud1-A308T* allele causes a weaker Cdc15 and downstream kinase activation phenotype than *CDC15-SPB*, since, in the former, Dbf2 is detected at the mSPB only during the metaphase. We ask the following question: if Cdc15 can localize to the SPBs in all cell cycle phases in *nud1-A308T* cells, what prevents Dbf2-Mob1 from becoming activated until metaphase? Most likely, this is due to inhibition by mitotic CDKs. Both Cdc15 and Mob1 are subject to inhibitory Clb/CDK phosphorylation [46,47]. Removal of Clb/CDK inhibition on Cdc15, using the *CDC15-7A* allele in metaphase-arrested cells, led to mitotic exit in a small percentage of cells where the spindle moved into the bud. Preventing mitotic CDK inhibition of Mob1 using the *MOB1-2A* allele had a comparatively much stronger effect [45]. It is possible that the alanine to threonine mutation in Nud1 causes a phenotype similar to *CDC15-7A* alone, where the inhibition on Cdc15 activity by Clb/CDKs is somehow bypassed by *nud1-A308T*, but the inhibitory phosphorylation of Mob1 remains. 

The localization of Dbf2-Mob1 to only the mSPB in metaphase, and then later to both dSPB and mSPB, is in agreement with prior work, which demonstrated that Dbf2-Mob1 localizes first to the mSPB in anaphase and then to the dSPB. In this way, the ultimate MEN kinase is distinct from Tem1 and Cdc15, which both accumulate at the dSPB before the mSPB [17]. The reason that Dbf2-Mob1 associates first with the mSPB in anaphase upon activation is not yet known, but the authors suggested that this could be due to lowered CDK inhibitory activity at the mSPB than at the dSPB. Therefore, we ask the following question: what could be occurring in the metaphase, which is, prior to the reduction in Clb/CDKs at the metaphase-to-anaphase transition, to allow Dbf2-Mob1 to localize to the mSPB early in mitosis, but not in prior cell cycle phases in *nud1-A308T* cells? It was recently shown that Cdc5 phosphorylation of Cfi1/Net1 in metaphase was important for Cdc14 activation in early and late anaphase [41]. However, we observed that the *NUD1* allele acts independently of *CDC5*. Therefore, this remains an interesting question that may shed light on further MEN-activating conditions during metaphase. 

A further question is as follows: does the *nud1-A308T* allele impact other SPB-mediated cellular processes? Nud1 is a critical component of the SPB inheritance network (SPIN), which causes the pre-existing “old” SPB to be inherited by the daughter cell, while the “new” SPB is inherited by the mSPB [55,56,57,58]. Specifically, Nud1 is modified by both the Swe1 kinase and NuA4 acetyltransferase complex and this allows the asymmetric segregation of the old SPB into the daughter cell, while the new SPB remains in the mother cell. This is due to the restriction of the spindle-positioning motor protein Kar9 to the old SPB in an MEN-dependent manner [56,57]. Interestingly, in mutants such as the *BFA1-SPC72* fusion and the GTPase-defective *TEM1-Q79L* allele, symmetric and premature loading of both the Tem1-GTPase and of Cdc15 led to increased symmetric Kar9 loading on both SPBs and resulting defects in mitotic spindle orientation and proper SPB inheritance [31,57]. Recent findings have suggested that constitutive disruption of proper SPB inheritance has negative consequences on replicative life span in budding yeast [59]. It will be interesting to see whether the early recruitment of Cdc15 and Dbf2 observed in the presence of *nud1-A308T* causes any alterations in SPB inheritance through the SPIN, and on aging. 

Our data leaves the possibility open, that the SPB component that Cdc15 binds to is the Nud1 MEN scaffold itself. An important question is the following: how does the mutation of alanine 308 to a threonine in Nud1 lead to Cdc5-independent recruitment of Cdc15 to SPBs? This is an important question because Cdc15 is homologous to PAK-kinases that are known to be effectors in Ras-GTPase cascades and contain an auto-inhibitory C-terminal domain [60]. Cdc15 is no different, since prior work demonstrated that deletion of its C-terminal domain led to constitutive localization of the protein to SPBs and hyperactivation of Dbf2-Mob1 activity [36,49]. One possibility is that the mutation creates a new phosphorylation site in Nud1 that leads to the recruitment of Cdc15 to the Nud1 scaffold. Nud1 is phosphorylated by the mitotic CDK Clb/Cdc28, so it is possible that a new docking site for Cdc15 is created by the mitotic kinase [61]. We do not favor this possibility; however, since the mutation does not create a site that fits consensus phosphorylation site for CDKs S/T-P-x-K/R (see Supplementary Figure S1). 

Another intriguing possibility is that the A308T alteration affects the stability of Nud1. It was demonstrated that Nud1 is subject to ubiquitin-mediated degradation in late mitosis by the E3 ubiquitin ligase Dma1/2. This overexpression of *DMA2* led to an inability to recruit Bfa1 and Mob1 to SPBs in anaphase, both of which are known to interact with Nud1. It also led to an inability to recruit Cdc15 to SPBs. The deletion of both *DMA1* and *DMA2* led to elevated levels of Bfa1, Cdc15, and Mob1 to SPBs in anaphase cells, while the tethering of Dma2 to SPBs led to cell death due to mitotic exit failure [62]. It is therefore possible that the mutation in *nud1-A308T* inhibits Dma1/2-mediated degradation, perhaps by preventing Dma1/2 association with Nud1 itself or with another SPB component, and this leads to recruitment of Cdc15 and subsequently Dbf2-Mob1 to Nud1. Interestingly, the sequence surrounding alanine 308 in Nud1 is highly conserved within the Saccharomyces clade, and weakly conserved in the mammalian homolog of Nud1, Centriolin (see Supplementary Figure S1). Centriolin is also required for late mitotic events, such as cytokinesis, and its loss can result in cytokinesis failure [63]. It is also important for proper mitotic spindle orientation during mammalian development, and loss of this protein from centrosomes is associated with severe developmental defects [64]. These findings highlight the possibility that further studies of the *nud1-A308T* allele may shed light on the regulation of Centriolin and the mechanisms by which normal embryonic development occurs in humans.

## Figures and Tables

**Table 1:** Strains used in this study. All strains are derivatives of W303 (AS3).

Number	Genotype
3	*MAT a ade2-1, leu2-3, ura3, trp1-1, his3-1115, can1-100, GAL, [phi+] (W303)*
5	*MAT a GAL-GFP-BFA1::HIS3MX6*
15	*MAT a TEM1-GFP:HIS3MX6*
53	*MAT a nud1-A308T*
79	*MAT a TEM1-GFP:HIS3MX6, HIS3MX6::GAL-BFA1*
112	*MAT a bub2::HIS3MX6*
138	*MAT a CDC14-3HA*
228	*MAT a cdc14-3, MOB1-6HA:HIS3MX6, NUD1-3V5::kanMX6*
296	*MAT a TEM1-GFP:HIS3MX6, HIS3MX6::GAL-BFA1, nud1(A308T)-3V5::kanMX6*
303	*MAT a nud1(A308T)-3V5::kanMX6, CDC14-3HA*
326	*MAT a NUD1-3V5::kanMX6*
383	*MAT a NUD1-3V5::kanMX6, trp1::YIP204-HIS3MX6::GAL-nud1(S53A,T78A)-3V5::TRP1*
384	*MAT a NUD1-3V5::kanMX6, trp1::YIP204-HIS3MX6::GAL-nud1(T78A)-3V5::TRP1*
385	*MAT a NUD1-3V5::kanMX6, trp1:: YIP204-HIS3MX6::GAL-nud1(S63A,T78A)-3V5::TRP1*
387	*MAT a nud1(A308T)-3V5::kanMX6*
388	*MAT a NUD1-3V5::kanMX6, trp1::YIP204-HIS3MX6::GAL-nud1(S53A,S63A,T78A)-3V5::TRP1*
390	*MAT a nud1(A308T)-3V5::kanMX6, trp1::YIP204-HIS3MX6::GAL-nud1(S53A,S63A,T78A)-3V5::TRP1*
392	*MAT a nud1(A308T)-3V5::kanMX6, trp1::YIP204-HIS3MX6::GAL-nud1(S63A,T78A)-3V5::TRP1*
395	*MAT a nud1(A308T)-3V5::kanMX6, trp1::YIP204-HIS3MX6::GAL-nud1(T78A)-3V5::TRP1*
397	*MAT a nud1(A308T)-3V5::kanMX6, trp1::YIP204-HIS3MX6::GAL-nud1(S53A,T78A)-3V5::TRP1*
402	*MAT a kanMX6::GAL-mob1-77*
407	*MAT a cdc14-3, MOB1-6HA:HIS3MX6*
417	*MAT a dyn1::HIS3MX6,* *ura3::pAFS125-TUB1p-GFP-TUB1::URA3*
439	*MAT a cdc14-3, MOB1-6HA:HIS3MX6, nud1(A308T)-3V5::kanMX6*
447	*MAT a cdc15-2, nud1(A308T)-3V5::kanMX6*
474	*MAT a dyn1::HIS3MX6,* *ura3::pAFS125-TUB1p-GFP-TUB1::URA3, nud1(A308T)-3V5::kanMX6*
476	*MAT a DBF2-eGFP::HIS3MX6, NUD1-3V5::kanMX6,* *ura3::pRS306-TUB1-mCherry-tub1::URA3*
477	*MAT a DBF2-eGFP::HIS3MX6, nud1(A308T)-3V5::kanMX6,* *ura3::pRS306-TUB1-mCherry-tub1::URA3*
478	*MAT a nud1(A308T)-3V5::kanMX6, kanMX6::GAL-mob1-77*
513	*MAT a CDC15-eGFP::kanMX6, NUD1-3V5::kanMX6,* *ura3::pRS306-TUB1-mCherry-tub1::URA3*
517	*MAT a CDC15-eGFP::kanMX6, nud1(A308T)-3V5::kanMX6,* *ura3::pRS306-TUB1-mCherry-tub1::URA3*
564	*MAT a cdc5-1*
570	*MAT a cdc5-1, nud1(A308T)-3V5::kanMX6*

## Supplementary Materials

The following are available online at https://www.mdpi.com/article/10.3390/cells11010046/s1, Figure S1: The mutant Nud1-A308T protein recruits Mob1 in both metaphase and anaphase cells—original blots.
