# Supplementary material for: A Novel Hyperactive Nud1 Mitotic Exit Network Scaffold Causes Spindle Position Checkpoint Bypass in Budding Yeast
Source: Cells. 2021 Dec 24;11(1):46. doi: 10.3390/cells11010046 (PMC8750578; doi:10.3390/cells11010046)
Supplement: Supplementary file 1 [file cells-11-00046-s001.zip › cells-1512780-supplementary.pdf]

Supplementary Material

The original blots for Figure 9 in the main text are shown below.

A.

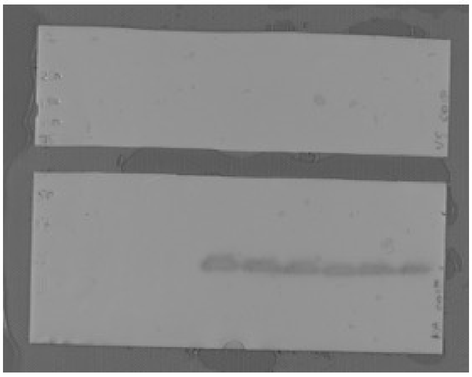

B.

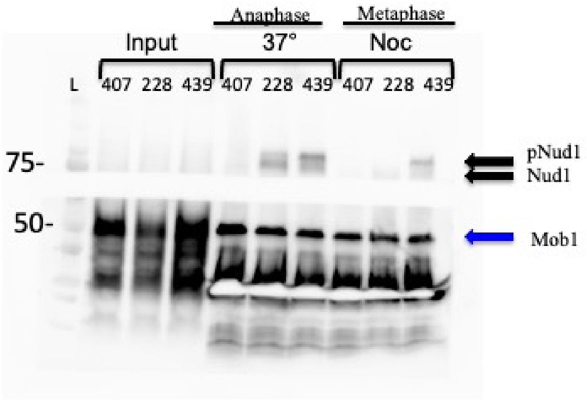

Figure S1. The mutant Nud1-A308T protein recruits Mob1 in both metaphase and anaphase cells—original blots.

MOB1-6HA (AS407), *MOB1-6HA NUD1-3V5* (AS228), and *MOB1-6HA nud1-A308T-V5* (AS439) cells harboring the *cdc14-3* mutation were grown to log phase. Cell cultures were split and half of each culture was incubated at 37°C for two hours to inactivate Cdc14 and produce an anaphase arrest (labeled anaphase). The other half of each culture was arrested with 15µg/mL nocodazole at 25°C to produce a metaphase arrest (labeled metaphase). Protein extracts were produced from each sample and Mob1-6HA was immunoprecipitated using anti-HA agarose beads. The samples were analyzed by SDS-PAGE and Western blotting using anti-HA antibody to detect the amount of Mob1-6HA immunopreciptated in each sample and anti-V5 antibodies to detect the amount of Nud1-3V5 or Nud1-A308T-3V5 co-immunoprecipitated. (**A**) The nitrocellulose filters are shown. The top filter was incubated with anti-V5 antibody to detect Nud1-3V5 or Nud1-A308T-3V5. The bottom filter was incubated with anti-HA antibody to detect Mob1-6HA. (**B**)**.** The input (100 µg) samples, anaphase samples and metaphase samples are labeled. Locations of Mob1 and Nud1/Nud1-A308T are labeled on the blot. Higher migrating phosphorylated species of Nud1 or Nud1-A308T are labeled (pNud1).
